# Physical activity and risk of venous thromboembolism: systematic review and meta-analysis of prospective cohort studies

**DOI:** 10.1007/s10654-019-00579-2

**Published:** 2019-11-14

**Authors:** Setor K. Kunutsor, Timo H. Mäkikallio, Samuel Seidu, Claudio Gil Soares de Araújo, Richard S. Dey, Ashley W. Blom, Jari A. Laukkanen

**Affiliations:** 1 National Institute for Health Research Bristol Biomedical Research Centre, University Hospitals Bristol NHS Foundation Trust and University of Bristol, Bristol, UK; 2 Musculoskeletal Research Unit, Translational Health Sciences, Bristol Medical School, University of Bristol, Learning and Research Building (Level 1), Southmead Hospital, Bristol, BS10 5NB UK; 3 Division of Cardiology, Department of Internal Medicine, Oulu University Hospital, Oulu, Finland; 4 Leicester Diabetes Centre, Leicester General Hospital, Gwendolen Road, Leicester, LE5 4WP UK; 5 Diabetes Research Centre, University of Leicester, Leicester General Hospital, Gwendolen Road, Leicester, LE5 4WP UK; 6 Exercise Medicine Clinic, Rio de Janeiro, Brazil; 7 University of Ghana Hospital, Legon, Ghana; 8 Institute of Public Health and Clinical Nutrition, University of Eastern Finland, Kuopio, Finland; 9 Faculty of Sport and Health Sciences, University of Jyväskylä, Jyvaskyla, Finland; 10 Department of Medicine, Central Finland Health Care District, Jyvaskyla, Finland

**Keywords:** Physical activity, Venous thromboembolism, Cohort study, Risk factor, Systematic review, Meta-analysis

## Abstract

The inverse association between physical activity and arterial thrombotic disease is well established. Evidence on the association between physical activity and venous thromboembolism (VTE) is divergent. We conducted a systematic review and meta-analysis of published observational prospective cohort studies evaluating the associations of physical activity with VTE risk. MEDLINE, Embase, Web of Science, and manual search of relevant bibliographies were systematically searched until 26 February 2019. Extracted relative risks (RRs) with 95% confidence intervals (CIs) for the maximum versus minimal amount of physical activity groups were pooled using random effects meta-analysis. Twelve articles based on 14 unique prospective cohort studies comprising of 1,286,295 participants and 23,753 VTE events were eligible. The pooled fully-adjusted RR (95% CI) of VTE comparing the most physically active versus the least physically active groups was 0.87 (0.79–0.95). In pooled analysis of 10 studies (288,043 participants and 7069 VTE events) that reported risk estimates not adjusted for body mass index (BMI), the RR (95% CI) of VTE was 0.81 (0.70–0.93). The associations did not vary by geographical location, age, sex, BMI, and methodological quality of studies. There was no evidence of publication bias among contributing studies. Pooled observational prospective cohort studies support an association between regular physical activity and low incidence of VTE. The relationship does not appear to be mediated or confounded by BMI.

**Electronic supplementary material:**

The online version of this article (10.1007/s10654-019-00579-2) contains supplementary material, which is available to authorized users.

## Introduction

Physical activity has several health benefits and its inverse and dose–response relationship with cardiovascular disease (CVD) (arterial thrombotic disease) is well established [1–3]. Physical activity may reduce vascular risk by exerting beneficial effects on physiological and metabolic processes through (1) improvements in endothelial function and levels of cardiovascular risk factors such as body weight, blood pressure, natriuretic peptides, lipid profiles, glucose tolerance, hemostatic factors, and cardiac troponin T [4–6]; (2) anti-inflammatory effects [7, 8]; and (3) enhancement of cardiac function [9, 10]. Venous thromboembolism (VTE) [comprising deep vein thrombosis (DVT) and pulmonary embolism (PE)], is closely linked with arterial thrombotic disease [11–13], and represents a growing public health burden due to increased morbidity, premature mortality, hospitalization, and associated healthcare costs [14–17]. The literature suggests both conditions may share some common risk factors such as obesity and cigarette smoking [18–20]. Factors implicated in the pathogenesis of VTE include inflammation, endothelial dysfunction, alterations in blood flow, immobilization, and hypercoagulable states [21–23] and physical activity is known to exert beneficial effects on some of these states [7, 24, 25]. Given the overall evidence, it is arguable that physical activity may lower the risk of VTE. Over the last decades, several studies have reported on the prospective associations of physical activity with the risk of VTE, but the results have been divergent. Some studies have reported decreased VTE risk with increased physical activity [26–28], whereas others have shown increased VTE risk with increased physical activity [29, 30] or no significant evidence of associations [31–33]. In a recent narrative review of existing observational prospective evidence, the authors evaluated and concluded that there may be a small beneficial effect of physical activity on the risk of incident VTE, but this did not appear to be consistent with a dose–response relationship [34]. A number of randomised controlled trials and observational studies have assessed the benefits and risks of physical activity on VTE, but these were based in patients or populations with acute or previous DVT [35]. A quantitative assessment of the association between physical activity and incident VTE using a systematic meta-analysis of the overall evidence has not yet been undertaken. Due to the wide uncertainty in the available evidence on this topic, we sought to evaluate in detail the prospective nature of the association between physical activity and future VTE risk using a systematic review and meta-analysis of all published observational prospective cohort studies conducted on the topic.

## Methods

### Data sources and searches

This systematic review and meta-analysis was registered in the PROSPERO prospective register of systematic reviews (CRD42019125869) and was conducted based on a predefined protocol and performed following PRISMA and MOOSE guidelines [36, 37] (Appendix 1–2 in ESM). We searched MEDLINE and Embase from inception to 26 February 2019. The computer-based searches used a combination of terms related to physical activity and VTE. There were no restrictions on language. Further details on the search strategy are presented in Appendix 3 in ESM. Titles and abstracts of studies retrieved from the databases were initially screened to assess their suitability for inclusion, after which we acquired potentially relevant articles for detailed full text evaluation. Two reviewers (SKK and SS) independently conducted full text evaluation using the inclusion criteria and any disagreements regarding eligibility of an article was discussed, and consensus reached with a third author (JAL). Reference lists of identified studies and relevant review articles were manually scanned and citing references were also checked in Web of Science, for additional eligible studies.

### Study selection

Observational population-based prospective (cohort, case cohort, or nested case–control) studies were eligible for inclusion if they had at least 1 year of follow-up and examined the relation of regular physical activity with the risk of first VTE in adult general populations. Case–control study designs were not included.

### Data extraction and quality assessment

One author (SKK) initially abstracted data from eligible studies using a standardized predesigned data collection form. A second reviewer (SS) independently checked these data with that in original articles. Any disagreements were discussed, and consensus reached with involvement of a third author (JAL). We extracted data on the following study characteristics: geographical location, period, design, participants (age, sex), sample size, duration of follow-up, assessment of physical activity, ascertainment of VTE case definition, number of participants developing VTE, and multivariate-adjusted relative risks (RRs), hazard ratios (HRs), or odds ratios (ORs) of VTE [and corresponding 95% confidence interval (CIs)]. Given emerging evidence that obesity (as measured using body mass index, BMI) is likely to reside in the causal pathway between physical activity and VTE, we also extracted data on the degree of adjustment for potential confounders (defined as ‘+’ minimally adjusted analysis, i.e. age and/or sex; ‘++’ as adjustment for established risk factors without BMI, i.e. age and/or sex plus cancer, socioeconomic status, smoking, or hypertension; and ‘+++’ as adjustment for established risk factors including BMI). To avoid double counting of a cohort, study selection was limited to a single set of most comprehensive results when there were multiple publications involving the same cohort. The priority for selection was the most up-to-date comprehensive study (longest follow-up or analysis covering the largest number of participants). We assessed the quality of studies on the basis of the nine-star Newcastle–Ottawa Scale (NOS) [38], which uses pre-defined criteria namely: selection (population representativeness), comparability (adjustment for confounders), and ascertainment of outcome. Nine points on the NOS reflects the highest study quality.

### Data synthesis and analysis

Summary measures were presented as RRs with 95% CIs. Following Cornfield’s rare disease assumption [39], HRs and ORs were assumed to approximate the same measure of RR. The majority of studies divided subjects into 2 or more groups on the basis of occupational physical activity, leisure-time physical activity, intensity of physical activity, or total or any physical activity. To enable a consistent approach to the meta-analysis and enhance comparison and interpretation of the findings, the extreme groups (i.e. maximum vs minimal amount of physical activity) were used for the analyses. When the highest physical activity group was the referent, we converted the reported risk estimate into its reciprocal. When a risk estimate was reported as a continuous measure (e.g., per unit change), this was transformed to a top versus bottom quantile using standard statistical methods [40] described previously [41]. When a study assessed types of physical activity in addition to total or any physical activity, we only used risk estimates for total or any physical activity in the pooled analysis. When studies published more than one estimate of the association according to subgroups (e.g., by sex), we obtained a within-study summary estimate using a fixed effect meta-analysis. To minimize the effect of between-study heterogeneity, RRs were pooled using a random effects model [42]. Quantification of the extent of statistical heterogeneity across studies employed standard Chi square tests and the I^2^ statistic [43, 44]. The 95% prediction intervals were also estimated to determine the degree of heterogeneity, as they provide a region in which about 95% of the true effects of a new study are expected to be found [45, 46]. To ensure the robustness of our findings, we performed sensitivity analyses by omitting studies (one at a time) that could have influenced the pooled RR (e.g., study with the largest sample size, study that assessed physical activity exposure retrospectively, and study with participants recruited from a clinical trial) and calculated a pooled estimate for the remainder of the studies. Study-level characteristics including geographical location, sex, average age at baseline, average duration of follow-up, number of cases, type of VTE, degree of adjustment (with or without adjustment for BMI), and study quality were pre-specified as characteristics for assessment of heterogeneity, which was conducted using stratified analysis and random effects meta-regression [47]. To assess the potential for publication bias, we constructed and visually inspected Begg’s funnel plots [48] and performed Egger’s regression symmetry test [49]. All analyses were conducted using Stata version 15 (Stata Corp, College Station, Texas).

## Results

### Study identification and selection

**Fig. 1:**
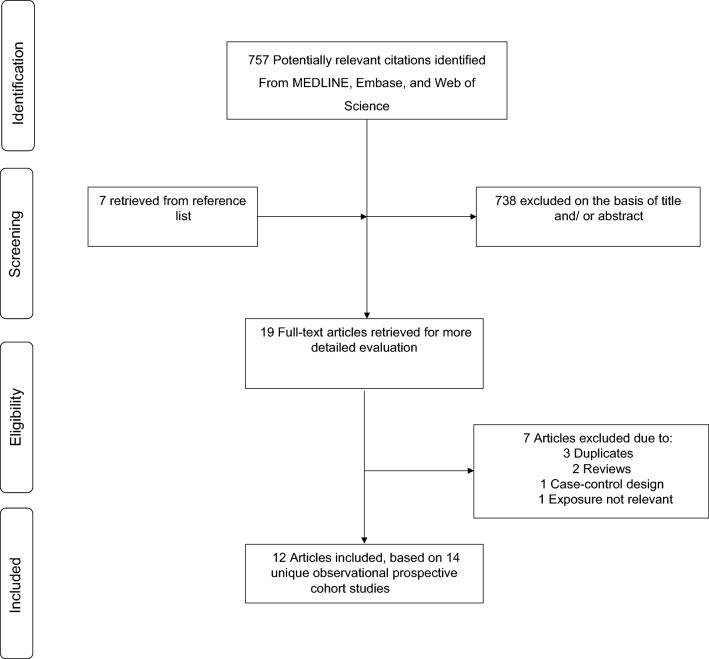
PRISMA flow diagram

Figure 1 illustrates the study selection process. Our initial search and manual screening of citations identified 757 potentially relevant citations. After screening of titles and abstracts, 19 articles remained for full text evaluation. We reviewed and excluded 7 articles because (1) they duplicated a previous publication using the same cohort (n = 3); (2) they were review articles (n = 2); (3) was a case–control design (n = 1); and (4) exposure was not relevant (n = 1) In total, we included 12 articles [26–30, 33, 50–55] based on 14 unique cohort studies comprising of 1,286,295 participants and 23,753 VTE events.

### Study characteristics and quality

Table 1 summarises characteristics of the 14 eligible studies on the association between physical activity and VTE. All 14 studies were based on prospective cohort study designs; however, one study was based on a prospective cohort follow-up of trial participants after the trial had been terminated [30]. For studies providing these data, the average age and BMI of participants at baseline ranged from approximately 46–65 years and 25.0–29.3 kg/m^2^ respectively; the weighted means were 52.1 years and 25.8 kg/m^2^ respectively. Four studies enrolled women only, two only men, and the rest enrolled both genders. Sample size of included cohorts ranged from 1766 to 992,228, with the number of VTE cases ranging from 171 to 14,550. The majority of studies (n = 9) included US populations, with five studies including European populations from Sweden, Norway, Denmark, and UK. Average duration of follow-up ranged from 5.0 to approximately 38.0 years, with a weighted mean of 9.6 years. All studies assessed physical activity through a questionnaire, but the categorisation of physical activity varied across studies. The diagnosis of VTE (DVT or PE) was based on a variety of methods which included duplex ultrasound, venogram, computed tomography, ventilation-perfusion scan, pulmonary angiogram, x-ray phlebography, and autopsy. Ascertainment of VTE was mostly based on self-reports, patient registries, hospital records, autopsy reports, or death registries. The degree of covariate adjustment varied, but majority of studies adjusted for risk factors such as age, sex, and BMI. Overall quality scores of studies ranged from 5 to 8.

**Table 1:** Characteristics of studies included in review (2005–2019)

Lead Author, Publication Date (Reference)	Name of study	Location	Population source	Year of baseline survey	Baseline age range (years)	% male	Average BMI (kg/m^2^)	Follow up (years)	Exposure	No. of VTE events	Total participants	Covariates adjusted for	Study quality
Glynn et al. [30]	PHS	USA	RCT^b^	1982–2003	40–84	100.0	NR	20.1	Exercise	358	18,662	None	6
van Stralen et al. [29]	CHS	USA	Population register	1989; 1992–1993	≥ 65	43.0	NR	11.6	Sports exercise	171	5534	Age, race, body mass index at baseline, and self-reported health as time-varying covariate	8
Lindqvist et al. [50]	MISS	Sweden	Population register	1990	25–64	0.0	NR	11.0	Regular exercise	312	29,518	Age, cancer, parity, smoking, alcohol, oral contraceptives, BMI	7
Holst et al. [33]	CCHS	Denmark	Population register	1976	≥ 20	46.0	25.0	19.5	Leisure-time PA	969	18,954	Age, calendar time	7
Lutsey et al. [51]	IWHS	USA	Driver’s license list	1986–2004	55–69	0.0	NR	13.0	PA	2137	40,377	Age, education, smoking, PA, BMI	7
Wattanakit et al. [26]	ARIC	USA	Population register	1987–1989	45–64	45.0	NR	15.5	PA	468	15,340	Age, race, ARIC field center, sex, BMI	7
Armstrong et al. [28]	Million Women Study	UK	National Health Service register	1996–2001	50–64	0.0	26.0	9.0	Any PA	14,550	992,228	BMI-by-age, smoking-by-age, alcohol-by-age, and stratified by socioeconomic status and region	7
Olson et al. [27]	REGARDS	USA	Population register	2003–2007	≥ 45	40.0	29.3	5.0	PA	263	30,239	Age, sex, income, education, race, region, race x region interaction	8
Ogunmoroti et al. [52]	MESA	USA	Population register	2000–2002	45–84	47.2	28.0	10.2	PA	215	6506	Age, sex, race/ethnicity, education, and income	7
Evensen et al. [53]	Tromso Study	Norway	Population register	1994–1995; 2001–2002; 2007–2008	25–89	47.6	25.3	6.8	PA	531	30,002	Age, sex, BMI, CVD, cancer	8
Kim et al. [54]	NHS	USA	Nurses register	1976	30–55	0.0	26.7	38.0	PA	889	2450	BMI and sitting time	5
Kim et al. [54]	NHS II	USA	Nurses register	1989	25–42	0.0	27.5	22.0	PA	447	1766	BMI and sitting time	5
Kim et al. [54]	HPFS	USA	Health Professionals register	1986	40–75	100.0	26.5	26.0	PA	798	1808	BMI and sitting time	5
Johansson et al. [55]	VEINS	Sweden	Population register	1985–2014	46.3^a^	NR	25.8	15.5	PA	1645	92,911	Age, body mass index, hypertension, smoking, education level and cancer	8

ARIC, Atherosclerosis Risk in Communities study; CHS, Cardiovascular Health Study; CCHS, Copenhagen City Heart Study; HPFS, Health Professionals Follow-up Study; IWHS, Iowa Women’s Health Study; MESA, Multi-Ethnic Study of Atherosclerosis; MISS, Melanoma Inquiry of Southern Sweden; NHS, Nurses’ Health Study; PHS, Physicians Health Study; REGARDS, Reasons for Geographic and Racial Differences in Stroke; VEINS, Venous thromboEmbolism In Northern Sweden; BMI, body mass index; CVD, cardiovascular disease; DVT, deep vein thrombosis; NR, not reported; PA, physical activity; PE, pulmonary embolism; VTE, venous thromboembolism

^a^Average age

^b^Based on a prospective cohort follow-up of trial participants after termination of trial

### Physical activity and incident VTE risk

**Fig. 2:**
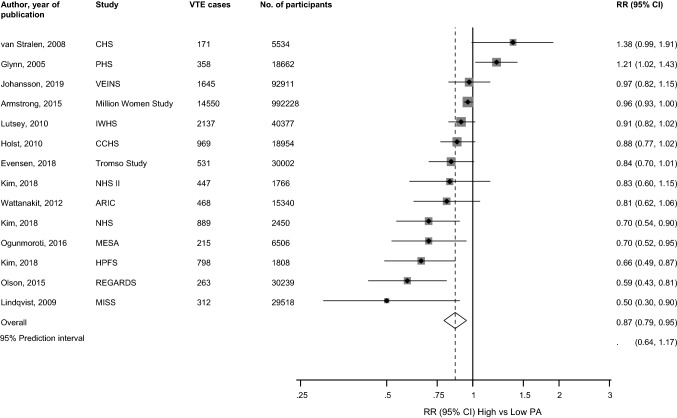
Prospective studies of physical activity and risk of venous thromboembolism included in meta-analysis. The summary estimate presented was calculated using random effects models and was based on fully adjusted estimates (including body mass index) where relevant; sizes of data markers are proportional to the inverse of the variance of the relative ratio; CI, confidence interval (bars); PA, physical activity; RR, relative risk; VTE, venous thromboembolism; study abbreviations are listed in Table 1

**Fig. 3:**
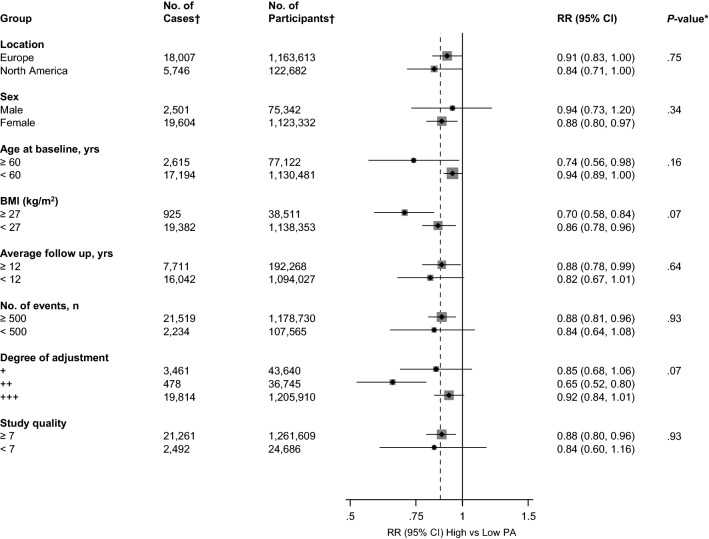
Relative risks for venous thromboembolism comparing maximal versus minimal amount of physical activity, grouped according to several study characteristics. The summary estimates presented were calculated using random effects models; CI, confidence interval (bars); PA, physical activity; RR, relative risk; VTE, venous thromboembolism; *, *p* value for meta-regression; **, defined as ‘+’ minimally adjusted analysis (age and/or sex); ‘++’ as adjustment for established risk factors without body mass index (age and/or sex plus cancer, socioeconomic status, smoking, or hypertension); and ‘+++’ as adjustment for established risk factors including body mass index; †, number of cases and participants are not equal across all the subgroups because not all studies reported data on these study characteristics

The pooled fully-adjusted (including BMI) RR (95% CI) of VTE comparing the most physically active versus the least physically active groups was 0.87 (0.79–0.95) (Fig. 2). The 95% prediction interval for the pooled RR was 0.64–1.17%, suggesting that the true RR for any single new study will usually fall within this range. There was evidence of heterogeneity between the contributing studies (*I*^2^= 73%, 53–84%; *p *< 0.001), which was not explained by any of the study level characteristics prespecified for subgroup analysis (Fig. 3). On exclusion of the study which reported physical activity as a continuous measure and was initially designed as a clinical trial [30], the RR (95% CI) of VTE comparing the most physically active versus the least physically active groups was 0.84 (0.77–0.92), with evidence of heterogeneity between the contributing studies (*I*^2^= 69%, 45–82%; *p *< 0.001). On exclusion of the largest study which was made up of only women [28], the RR (95% CI) of VTE was 0.85 (0.75–0.95) with heterogeneity of (*I*^2^= 72%, 51–83%; *p *< 0.001). Furthermore, on exclusion of the study which assessed physical activity retrospectively [50], the RR (95% CI) of VTE was 0.88 (0.80–0.96) with heterogeneity of (*I*^2^= 72%, 51–84%; *p *< 0.001).

In pooled analysis of 10 studies (288,043 participants and 7069 VTE events) that reported risk estimates not adjusted for BMI, the RR (95% CI) of VTE was 0.81 (0.70–0.93) (Appendix 4 in ESM).

### Publication bias

A funnel plot of the 14 studies reporting on the associations between physical activity and VTE risk showed visual evidence of symmetry (Appendix 5 in ESM) which was consistent with Egger’s regression symmetry test (*p *= 0.065). We also found no evidence of such selective reporting when studies were grouped by size in meta-regression analysis (Fig. 3).

## Comments

### Summary of main findings

Given the uncertain evidence on the prospective relationship between physical activity and VTE risk as well as the absence of any quantitative evidence summarizing the existing literature, we have conducted the first meta-analysis of population-based prospective cohort studies to evaluate the association between regular physical activity and VTE. In pooled analysis of 14 studies, regular physical activity was significantly associated with a lower risk of VTE when compared with a sedentary or less active lifestyle. In contrast to previous studies, the association did not appear to be mediated or confounded by BMI. Furthermore, the inverse association between physical activity and VTE risk was not modified by geographical location, sex, age, duration of follow-up, degree of adjustment, and methodological quality of studies.

### Comparison with previous work

To the best of our knowledge, there have been no previous efforts to aggregate existing data on the relation between physical activity and future VTE risk quantitatively. Being the first comprehensive meta-analysis on the topic, the findings cannot be directly compared to previous work. Though there has been overwhelming evidence showing that regular physical activity is associated with reduced risk of arterial thrombotic disease [1–3], findings on the relationship between physical activity and VTE risk have been mixed in the absence of a pooled analysis. In a recently published narrative review that evaluated the existing epidemiological evidence on the association between physical activity and VTE risk, Evensen and colleagues concluded that there might be a modest beneficial effect of physical activity on incident VTE risk [34]. However, the evidence was hampered by the variance in the assessment and definition of the exposure variable—physical activity. By combining the available evidence in a systematic and quantitative manner, we have shown that regular physical activity is also associated with a reduced risk of VTE compared with physical inactivity. In contrast to evidence from some individual studies [26, 53], the association did not appear to be mediated or confounded by BMI. Though there was no statistically significant evidence of effect modification by age on the association, there was a suggestion that the beneficial effect of physical activity on VTE risk was stronger in the elderly, a finding which is consistent with previous evidence [53].

### Possible explanations for findings

To date, obesity and smoking have consistently been demonstrated to be associated with VTE risk [18–20, 26]. The current findings suggest that another common lifestyle factor shared by VTE and atherosclerotic CVD could be physical inactivity. Both disease states share common characteristics such as coagulation and platelet activation, hence they may have common pathophysiological mechanisms [56]. On the contrary, atherosclerotic CVD and VTE have historically been viewed as two distinct diseases [57] and based on findings that traditional risk factors for VTE and arterial thrombotic disease are not similar, it is generally believed that their pathogenesis differ [31, 58]. The pathophysiological mechanisms underlying the association between regular physical activity and reduced VTE risk may relate to the ability of physical activity to (1) improve levels of potential risk factors such as body weight, hypertension, and lipids; [59] (2) decrease systemic inflammation; [7, 8] and (3) decrease plasma viscosity [25] and platelet aggregation [60], all of which are involved in VTE pathophysiology. Increased muscular activity of the lower limbs as a result of regular physical activity could also increase venous return and decrease VTE risk [61]. Mechanistic conclusions underlying the association between physical activity and VTE cannot be drawn from observational epidemiological studies and need further specific studies to clarify these pathways.

### Implications of findings

The potential association between regular physical activity and decreased VTE risk may have clinical implications with respect to VTE prevention. Just like CVD, physical activity may represent an important approach for VTE prevention, for instance in the areas of screening of individuals at risk of VTE, recommending lifestyle modification, as well as further management. Though there is no clinical trial evidence showing regular physical activity can reduce the incidence of VTE, RCT evidence shows physical activity to be associated with reduced severity of VTE complications such as post thrombotic syndrome [35]. The health benefits associated with regular PA, cannot be overemphasized. The Physical Activity Guidelines Advisory Committee Scientific Report recommends 150–300 min/week of moderate-intensity or 75–150 min/week of vigorous-intensity aerobic PA/exercise for adults, as this is associated with substantial health benefits in most people; [62] however, a substantial proportion of the population do not achieve these recommended levels. For example in the United States, only 46% of adults meet the general physical activity recommendations [63]. Though there are still some unanswered questions such as the nature of the dose–response relationship between physical activity and VTE risk and the optimal physical activity intensity, frequency, and duration for VTE prevention, it is recommended that physically inactive adults should engage in some regular physical activity to improve their overall vascular health. Even standing, which is the least active behaviour, has been reported to be associated with health benefits compared with sitting [64].

### Strengths and limitations

Given the inconsistent evidence on the topic, this study represents the first attempt at summarising the overall evidence using a systematic meta-analysis. We employed a comprehensive search strategy across multiple databases with no language restrictions and undertook manual reference scanning; which made it unlikely that we had missed any relevant study conducted on the topic. Other strengths were the comprehensive analyses which included exploration of heterogeneity using stratification by several study level characteristics and several sensitivity analyses. Finally, formal tests showed no evidence of publication bias or selective reporting. However, despite the lack of strong evidence of publication bias, we cannot completely rule out the influence of selective reporting; since tests for publication bias have low statistical power. There were important limitations to this review and these were all inherent to the included studies. The included studies examined different types of physical activity and categorised the amount of physical activity differently, hence comparisons could only be made between the most and least active and there was difficulty in combining data across studies to assess a dose–response relation between physical activity and VTE. The effect of the different types/modalities of physical activity could not be explored because of the limited number of studies consistently reporting on the same type of physical activity. There was the potential for misclassification bias because physical activity was self-reported and its classification was study-specific. Studies did not report risk estimates for the specific endpoints of DVT and PE and therefore their associations could not be evaluated. There was substantial heterogeneity between contributing studies which could not be explained by several clinically relevant study level characteristics, suggesting that other factors might be at play. One study based on only women contributed about 77% of the overall sample size, however, exclusion of this study in sensitivity analysis did not change the overall estimate. Pooled analysis was based on variably adjusted data reported by the eligible studies, therefore prone to confounding by unmeasured factors. Finally, estimated prediction intervals of the pooled RRs of the associations contained values on both sides of the null and so, although on average there was evidence of an association of regular physical activity with VTE risk, this may not always be the case in other studies. The findings should therefore be interpreted with caution given these limitations. To address the issues with standardization of physical activity, consistent adjustment for confounding, exploration of dose–response relationships and assessment of heterogeneity, we propose an individual participant data meta-analysis of these prospective cohort studies.

## Conclusion

New evidence based on a comprehensive meta-analysis of all observational prospective cohort studies support an association between regular physical activity and low incidence of VTE. In contrast to previous evidence, the potential protective effect of physical activity on VTE does not appear to be mediated or confounded by BMI. These findings should stimulate further efforts to further clarify the relationship between physical activity and VTE risk.

## Electronic supplementary material

Below is the link to the electronic supplementary material.

Supplementary material 1 (DOC 402 kb)
