# Supplementary material for: Physical activity and risk of venous thromboembolism: systematic review and meta-analysis of prospective cohort studies
Source: Eur J Epidemiol. 2019 Nov 14;35(5):431–42. doi: 10.1007/s10654-019-00579-2 (PMC7250794; doi:10.1007/s10654-019-00579-2)
Supplement: Supplementary file 1 — Supplementary material 1 (DOC 402 kb) [file 10654_2019_579_MOESM1_ESM.doc]

**Supplementary Material**

| **Appendix 1** | PRISMA checklist |
| --- | --- |
| **Appendix 2** | MOOSE checklist |
| **Appendix 3** | Literature search strategy |
| **Appendix 4** | Relative risks for venous thromboembolism comparing maximal versus minimal amount of physical activity in models not adjusted for body mass index |
| **Appendix 5** | Assessment of small study effects by funnel plots and Egger’s regression symmetry tests |

**Appendix 1.** PRISMA checklist

| **Section/topic** | **Item No** | **Checklist item** | **Reported on page No** |
| --- | --- | --- | --- |
| **Title** | | | |
| Title | 1 | Identify the report as a systematic review, meta-analysis, or both | 1 |
| **Abstract** | | | |
| Structured summary | 2 | Provide a structured summary including, as applicable, background, objectives, data sources, study eligibility criteria, participants, interventions, study appraisal and synthesis methods, results, limitations, conclusions and implications of key findings, systematic review registration number | 2 |
| **Introduction** | | | |
| Rationale | 3 | Describe the rationale for the review in the context of what is already known | 3 |
| Objectives | 4 | Provide an explicit statement of questions being addressed with reference to participants, interventions, comparisons, outcomes, and study design (PICOS) | 3 |
| **Methods** | | | |
| Protocol and registration | 5 | Indicate if a review protocol exists, if and where it can be accessed (such as web address), and, if available, provide registration information including registration number | 4 |
| Eligibility criteria | 6 | Specify study characteristics (such as PICOS, length of follow-up) and report characteristics (such as years considered, language, publication status) used as criteria for eligibility, giving rationale | 4 |
| Information sources | 7 | Describe all information sources (such as databases with dates of coverage, contact with study authors to identify additional studies) in the search and date last searched | 4 |
| Search | 8 | Present full electronic search strategy for at least one database, including any limits used, such that it could be repeated | Appendix 3 |
| Study selection | 9 | State the process for selecting studies (that is, screening, eligibility, included in systematic review, and, if applicable, included in the meta-analysis) | 4-5 |
| Data collection process | 10 | Describe method of data extraction from reports (such as piloted forms, independently, in duplicate) and any processes for obtaining and confirming data from investigators | 4-5 |
| Data items | 11 | List and define all variables for which data were sought (such as PICOS, funding sources) and any assumptions and simplifications made | 4-5 |
| Risk of bias in individual studies | 12 | Describe methods used for assessing risk of bias of individual studies (including specification of whether this was done at the study or outcome level), and how this information is to be used in any data synthesis | 4-5 |
| Summary measures | 13 | State the principal summary measures (such as risk ratio, difference in means). | 5-6 |
| Synthesis of results | 14 | Describe the methods of handling data and combining results of studies, if done, including measures of consistency (such as I^2^ statistic) for each meta-analysis | 5-6 |
| Risk of bias across studies | 15 | Specify any assessment of risk of bias that may affect the cumulative evidence (such as publication bias, selective reporting within studies) | 5-6 |
| Additional analyses | 16 | Describe methods of additional analyses (such as sensitivity or subgroup analyses, meta-regression), if done, indicating which were pre-specified | 5-6 |
| **Results** | | | |
| Study selection | 17 | Give numbers of studies screened, assessed for eligibility, and included in the review, with reasons for exclusions at each stage, ideally with a flow diagram | 6 and Figure 1 |
| Study characteristics | 18 | For each study, present characteristics for which data were extracted (such as study size, PICOS, follow-up period) and provide the citations | 7, Table 1 |
| Risk of bias within studies | 19 | Present data on risk of bias of each study and, if available, any outcome-level assessment (see item 12). | 7, Table 1 |
| Results of individual studies | 20 | For all outcomes considered (benefits or harms), present for each study (a) simple summary data for each intervention group and (b) effect estimates and confidence intervals, ideally with a forest plot | 7-8, Figure 2 |
| Synthesis of results | 21 | Present results of each meta-analysis done, including confidence intervals and measures of consistency | 7-8, Figure 2 |
| Risk of bias across studies | 22 | Present results of any assessment of risk of bias across studies (see item 15) | Figure 3 |
| Additional analysis | 23 | Give results of additional analyses, if done (such as sensitivity or subgroup analyses, meta-regression) (see item 16) | 7-8; Figure 3; Appendices 4-5 |
| **Discussion** | | | |
| Summary of evidence | 24 | Summarise the main findings including the strength of evidence for each main outcome; consider their relevance to key groups (such as health care providers, users, and policy makers) | 8 |
| Limitations | 25 | Discuss limitations at study and outcome level (such as risk of bias), and at review level (such as incomplete retrieval of identified research, reporting bias) | 11-12 |
| Conclusions | 26 | Provide a general interpretation of the results in the context of other evidence, and implications for future research | 12 |
| **Funding** | | | |
| Funding | 27 | Describe sources of funding for the systematic review and other support (such as supply of data) and role of funders for the systematic review | 12-13 |

**Appendix 2.** MOOSE checklist

**Physical activity and risk of venous thromboembolism: systematic review and meta-analysis of prospective cohort studies**

| **Criteria** | | **Brief description of how the criteria were handled in the review** |
| --- | --- | --- |
| **Reporting of background** | |  |
| √ | Problem definition | The inverse association between physical activity and arterial thrombotic disease is well established. Evidence on the association between physical activity and venous thromboembolism (VTE) is not consistent. |
| √ | Hypothesis statement | Regular physical activity is associated with reduced risk of VTE. |
| √ | Description of study outcomes | VTE (pulmonary embolism and deep vein thrombosis |
| √ | Type of exposure | Physical activity |
| √ | Type of study designs used | Prospective cohort studies |
| √ | Study population | Adult general populations with assessment of physical activity at study entry |
| **Reporting of search strategy should include** | |  |
| √ | Qualifications of searchers | Setor K. Kunutsor, PhD; Richard S. Dey, MD; Samuel Seidu, MD |
| √ | Search strategy, including time period included in the synthesis and keywords | Time period: from inception to 26 February 2019  The detailed search strategy can be found in Appendix 3 |
| √ | Databases and registries searched | MEDLINE, Embase, Web of Science |
| √ | Search software used, name and version, including special features | OvidSP was used to search Embase and MEDLINE  EndNote X9 used to manage references |
| √ | Use of hand searching | We searched bibliographies of retrieved papers |
| √ | List of citations located and those excluded, including justifications | Details of the literature search process are outlined in the flow chart. The citation list for excluded studies are available on request. |
| √ | Method of addressing articles published in languages other than English | Not applicable |
| √ | Method of handling abstracts and unpublished studies | Abstracts with no full text publications were not included. |
| √ | Description of any contact with authors | None |
| **Reporting of methods should include** | |  |
| √ | Description of relevance or appropriateness of studies assembled for assessing the hypothesis to be tested | Detailed inclusion and exclusion criteria are described in the Methods section. |
| √ | Rationale for the selection and coding of data | Data extracted from each of the studies were relevant to the population characteristics, study design, exposure, and outcome. |
| √ | Assessment of confounding | We assessed confounding by ranking individual studies on the basis of different adjustment levels and performed sub-group analyses to evaluate differences in the overall estimates according to levels of adjustment. |
| √ | Assessment of study quality, including blinding of quality assessors; stratification or regression on possible predictors of study results | Study quality was assessed based on the nine-star Newcastle–Ottawa Scale using pre-defined criteria namely: population representativeness, comparability (adjustment of confounders), ascertainment of outcome. Sensitivity analyses by several quality indicators such as study size, duration of follow-up, and adjustment factors. |
| √ | Assessment of heterogeneity | Heterogeneity of the studies was quantified with I^2^ statistic that provides the relative amount of variance of the summary effect due to the between-study heterogeneity and explored using meta-regression and stratified analyses |
| √ | Description of statistical methods in sufficient detail to be replicated | Description of methods of meta-analyses, sensitivity analyses, meta-regression and assessment of publication bias are detailed in the methods. We performed random effects meta-analysis with Stata 15. |
| √ | Provision of appropriate tables and graphics | Table 1; Figures 1-3; Appendices 4-5 |
| **Reporting of results should include** | |  |
| √ | Graph summarizing individual study estimates and overall estimate | Figure 2 |
| √ | Table giving descriptive information for each study included | Table 1 |
| √ | Results of sensitivity testing | Sensitivity analysis was conducted to assess the influence of some large studies and low-quality studies on the pooled estimate. |
| √ | Indication of statistical uncertainty of findings | 95% confidence intervals were presented with all summary estimates, I^2^ values and results of sensitivity analyses |
| **Reporting of discussion should include** | |  |
| √ | Quantitative assessment of bias | Sensitivity analyses indicate heterogeneity in strengths of the association due to most common biases in observational studies. The systematic review is limited in scope, as it involves published data. Individual participant data is needed. Limitations have been discussed. |
| √ | Justification for exclusion | All studies were excluded based on the pre-defined inclusion criteria in methods section. |
| √ | Assessment of quality of included studies | Brief discussion included in ‘Methods’ section |
| **Reporting of conclusions should include** | |  |
| √ | Consideration of alternative explanations for observed results | Discussion |
| √ | Generalization of the conclusions | Discussed in the context of the results. |
| √ | Guidelines for future research | We recommend individual participant data meta-analysis |
| √ | Disclosure of funding source | In “Acknowledgement” section |

**Appendix 3.** Literature search strategy

Relevant studies, published from inception to 26 February 2019 (date last searched), were identified through electronic searches limited to the English language using MEDLINE, Embase, and Web of Science databases. Electronic searches were supplemented by scanning reference lists of articles identified for all relevant studies (including review articles) and by hand searching of relevant journals.

| Database: Ovid MEDLINE(R) <1946 to present>  Search Strategy:  --------------------------------------------------------------------------------  1 exp Exercise/ (174950)  2 physical activity.mp. (93175)  3 physical inactivity.mp. (6744)  4 exp Venous Thrombosis/ (52609)  5 venous thrombus.mp. (525)  6 exp Venous Thromboembolism/ (9031)  7 exp Pulmonary Embolism/ (37035)  8 deep vein thrombosis.mp. (15286)  9 1 or 2 or 3 (236421)  10 4 or 5 or 6 or 7 or 8 (93740)  11 9 and 10 (335)  12 limit 11 to humans (327)  ***************************  Each part was specifically translated for searching the other databases (Embase and Web of Science) |
| --- |

**Appendix 4.** Relative risks for venous thromboembolism comparing maximal versus minimal amount of physical activity in models not adjusted for body mass index

The summary estimate presented was calculated using random effects models; sizes of data markers are proportional to the inverse of the variance of the relative ratio; CI, confidence interval (bars); PA, physical activity; RR, relative risk; VTE, venous thromboembolism; Study abbreviations are listed in full in Table 1.

**Appendix 5.** Assessment of small study effects by funnel plots and Egger’s regression symmetry tests
